# Publisher Correction: An integrated high-throughput robotic platform and active learning approach for accelerated discovery of optimal electrolyte formulations

**DOI:** 10.1038/s41467-024-47608-7

**Published:** 2024-04-11

**Authors:** Juran Noh, Hieu A. Doan, Heather Job, Lily A. Robertson, Lu Zhang, Rajeev S. Assary, Karl Mueller, Vijayakumar Murugesan, Yangang Liang

**Affiliations:** 1 Energy and Environment Directorate, Pacific Northwest National Laboratory, Richland, WA 99354 USA; 2 Materials Science Division, Argonne National Laboratory, Lemont, IL 60439 USA; 3 Chemical Sciences and Engineering Division, Argonne National Laboratory, Lemont, IL 60439 USA; 4 Physical and Computational Sciences Directorate, Pacific Northwest National Laboratory, Richland, WA 99354 USA

**Keywords:** Batteries, Batteries, Energy

Correction to: *Nature Communications* 10.1038/s41467-024-47070-5, published online 29 March 2024

The copyright holder for this article was incorrectly given as ‘This is a U.S. Government work and not under copyright protection in the US; foreign copyright protection may apply’ but should have been ‘Battelle Memorial Institute & Argonne National Laboratory’.

