# Supplementary material for: An integrated high-throughput robotic platform and active learning approach for accelerated discovery of optimal electrolyte formulations
Source: Nat Commun. 2024 Mar 29;15:2757. doi: 10.1038/s41467-024-47070-5 (PMC10980761; doi:10.1038/s41467-024-47070-5)
Supplement: Supplementary file 1 — Supplementary Information [file 41467_2024_47070_MOESM1_ESM.pdf]

Supplementary Information for

An Integrated High-throughput Robotic Platform and Active Learning Approach for Accelerated Discovery of Optimal Electrolyte Formulations

Juran Noh[1,5], Hieu A. Doan[2,5], Heather Job[1], Lily A. Robertson[3], Lu Zhang[3], Rajeev S. Assary[2], Karl Mueller[4], Vijayakumar Murugesan,[4*] and Yangang Liang[1*]*

[1]Energy and Environment Directorate, Pacific Northwest National Laboratory, Richland, Washington 99354, USA.

[2]Materials Science Division, Argonne National Laboratory, Lemont, Illinois 60439, USA.

[3]Chemical Sciences and Engineering Division, Argonne National Laboratory, Lemont, Illinois 60439, USA.

[4]Physical and Computational Sciences Directorate, Pacific Northwest National Laboratory, Richland, Washington 99354, USA.

[5]These authors contributed equally: J. Noh and H. A. Doan

[*]Email: hadoan@anl.gov, vijay@pnnl.gov, yangang.liang@pnnl.gov

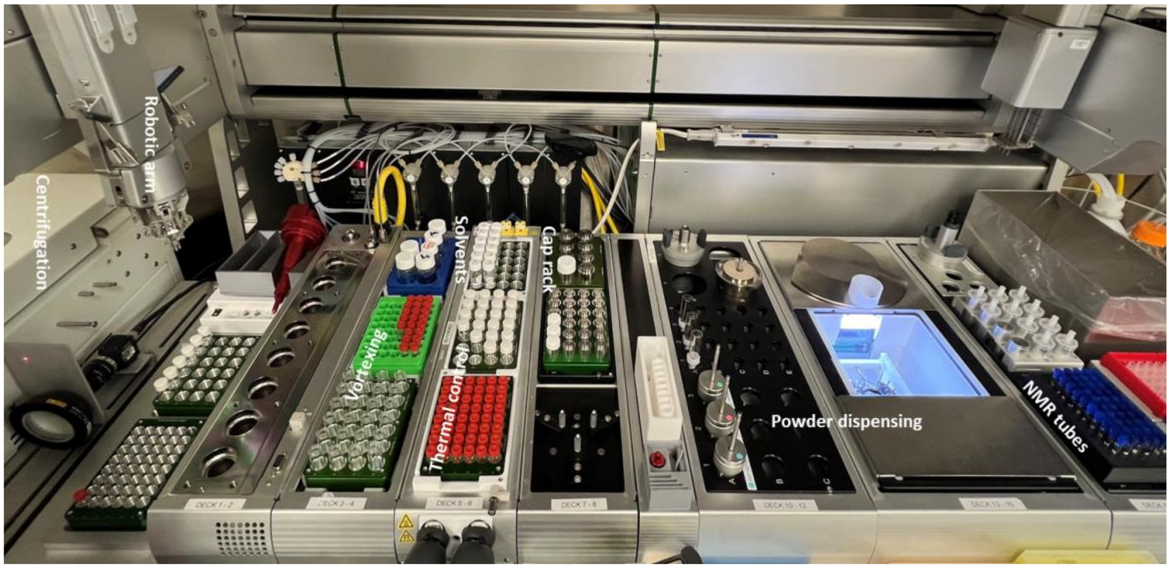

Fig. S1. Photograph of our automated HTE robotic platform for sample preparation.

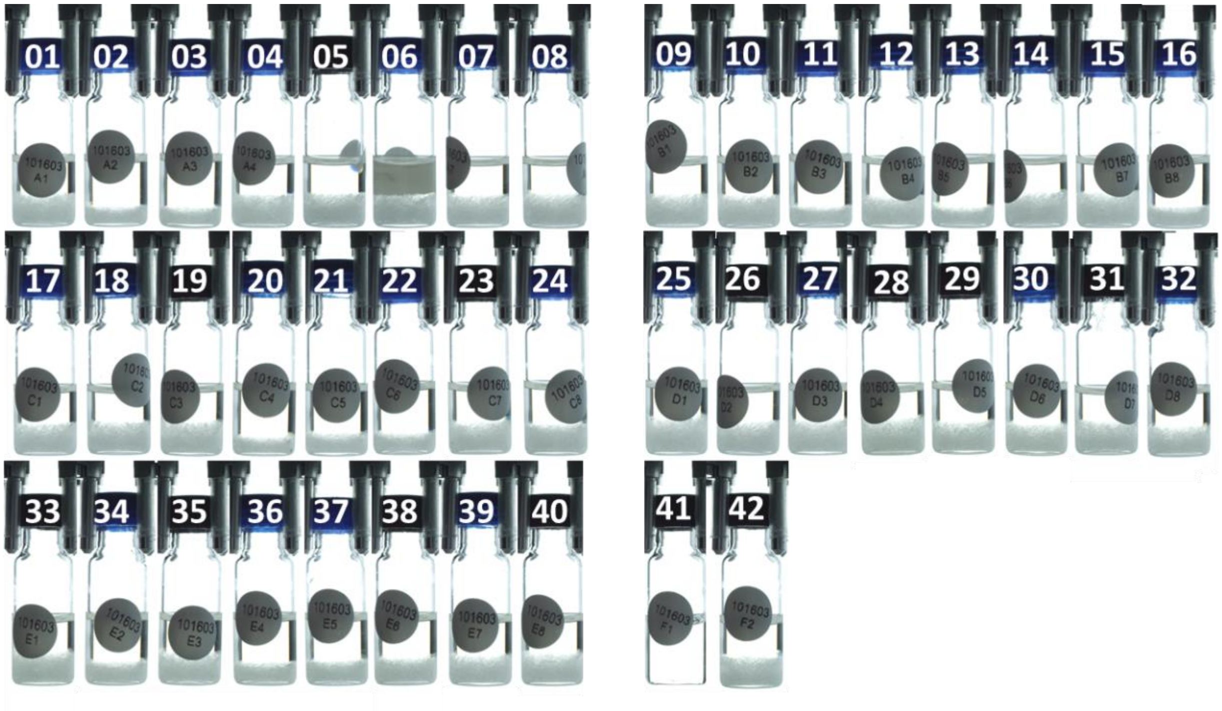

Fig. S2. Photograph of saturated solutions prior to solubility measurement.

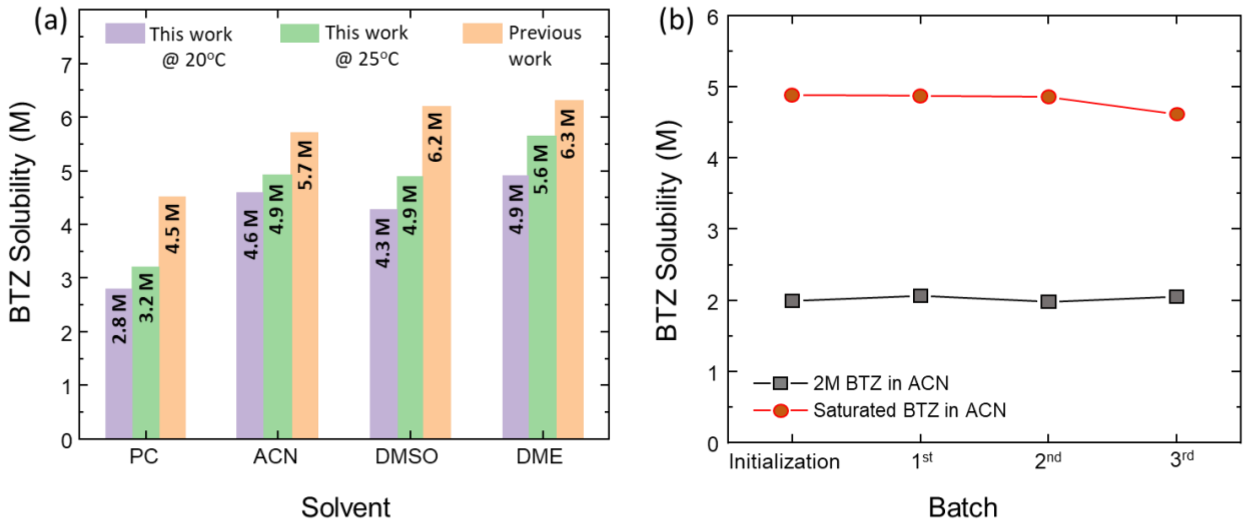

Fig. S3. (a) Measured solubilities of BTZ in various solvents in previous (*ACS Energy Letters 2, 1156-1161 (2017)*) and current work. (b) Concentrations of BTZ in two control samples across various batches. Source data are provided as a Source Data file.

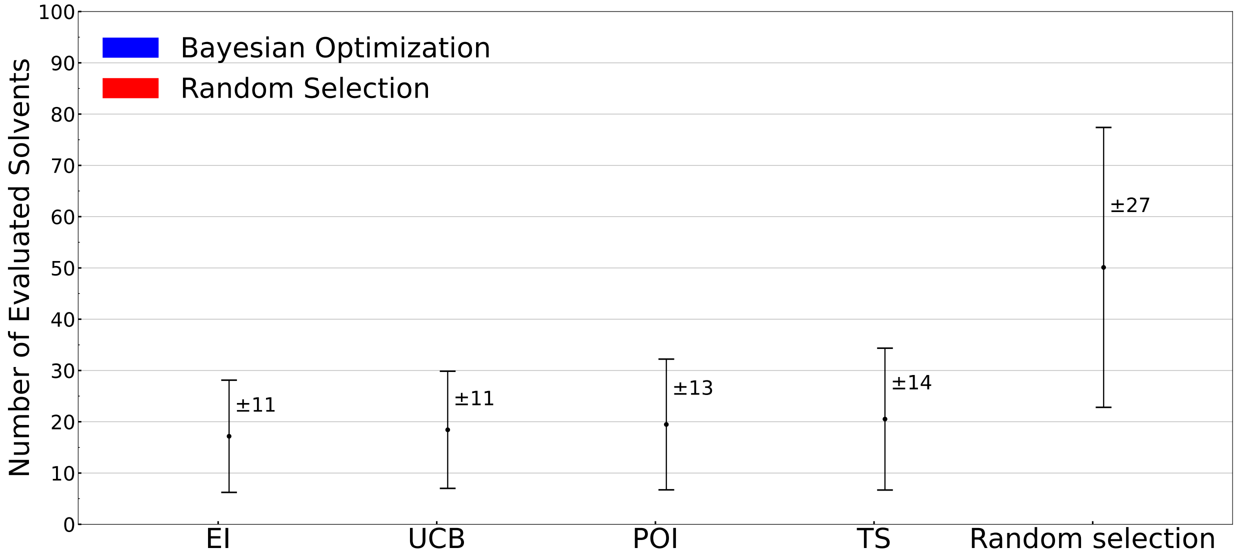

Fig. S4. Comparison of random selection versus Bayesian optimization using various acquisitions on the initial known dataset of 98 solvents. The height of the color bar and black error bar represent the mean and standard deviation of 100 trials. EI, UCB, POI, and TS stand for expected improvement, upper confidence bound, probability of improvement, and Thompson sampling. Source data are provided as a Source Data file.

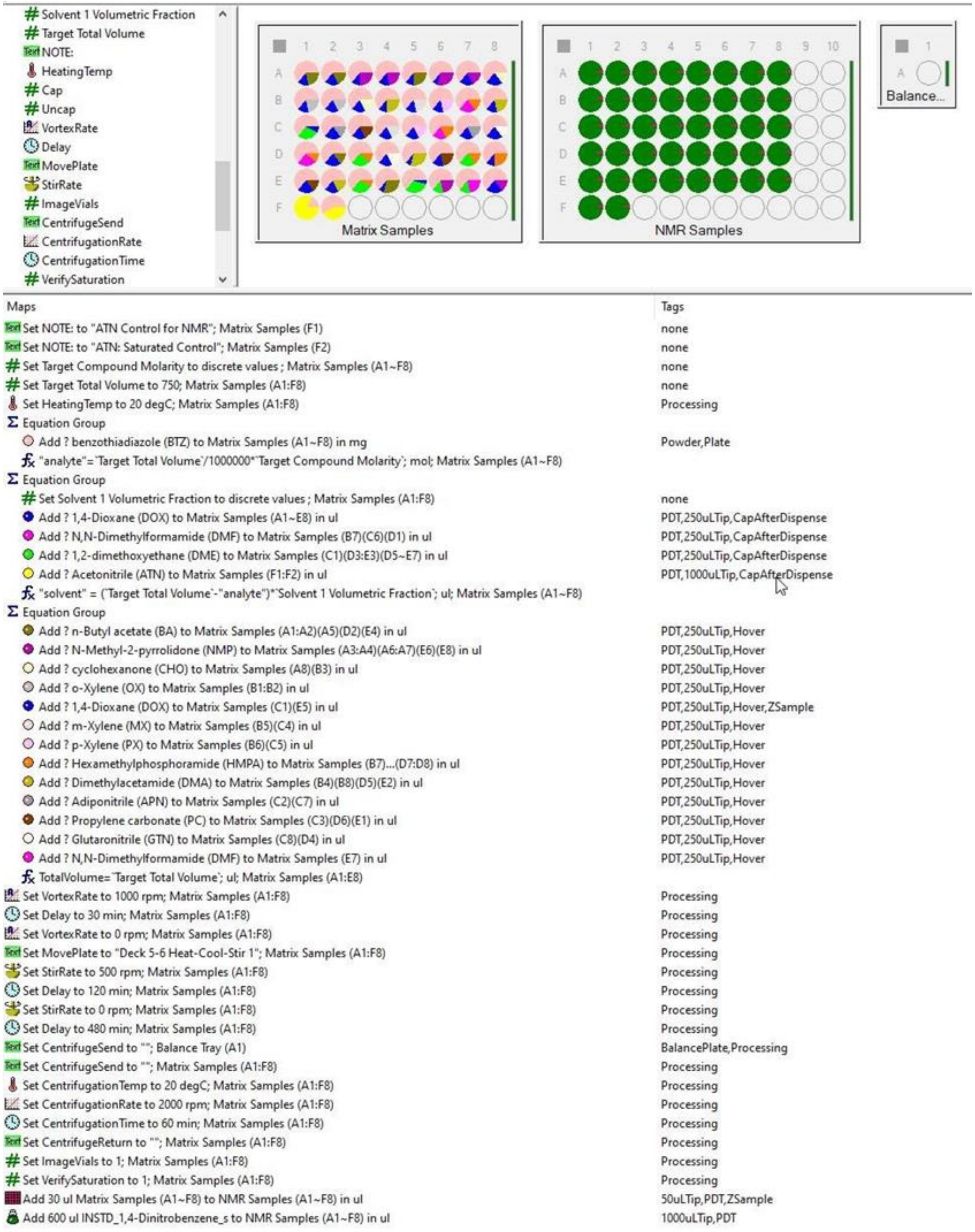

Fig. S5. The workflow of the designed sampling process on the robotic platform.

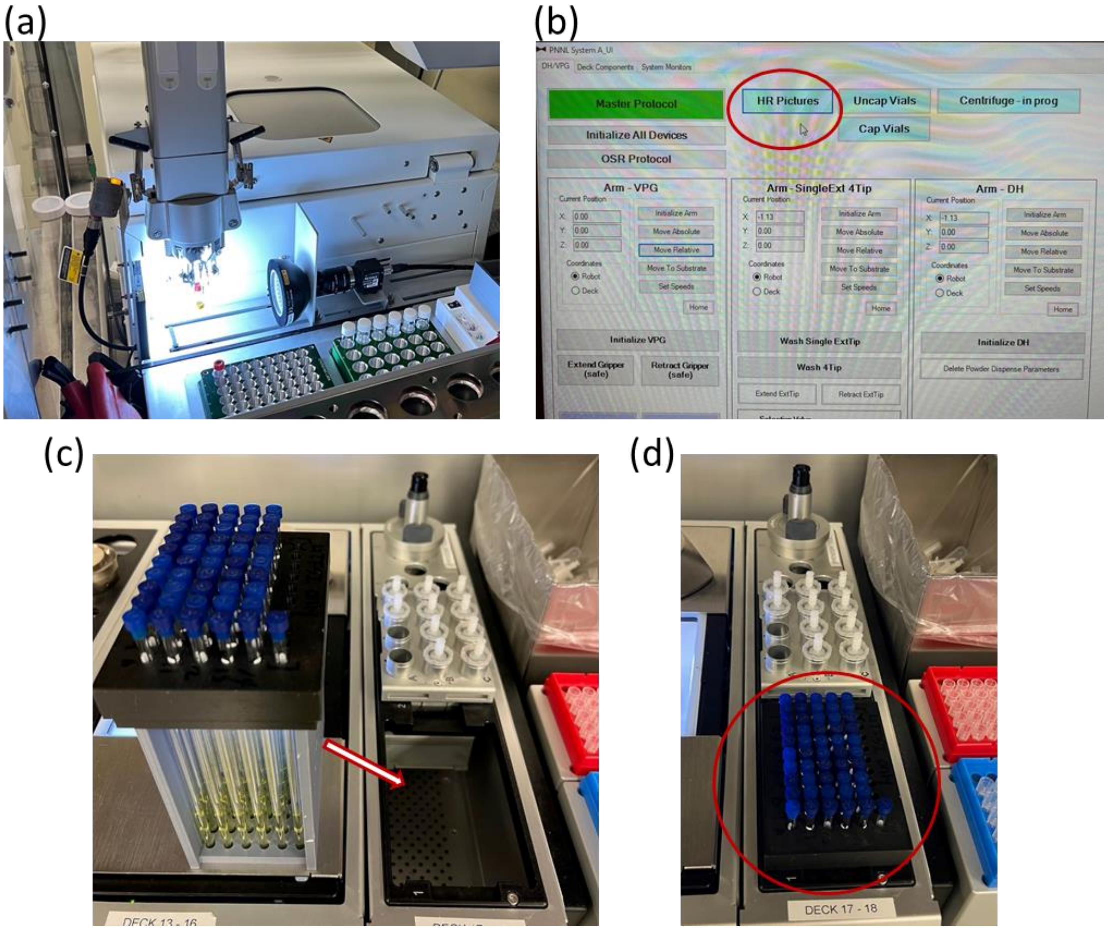

Fig. S6. Integration of (a) Keyence's CV-X vision system and (b) its control software in the operating system of our robotic platform. (c) Stand-alone and (d) on-deck picture of the 3D-printed NMR tube holder.

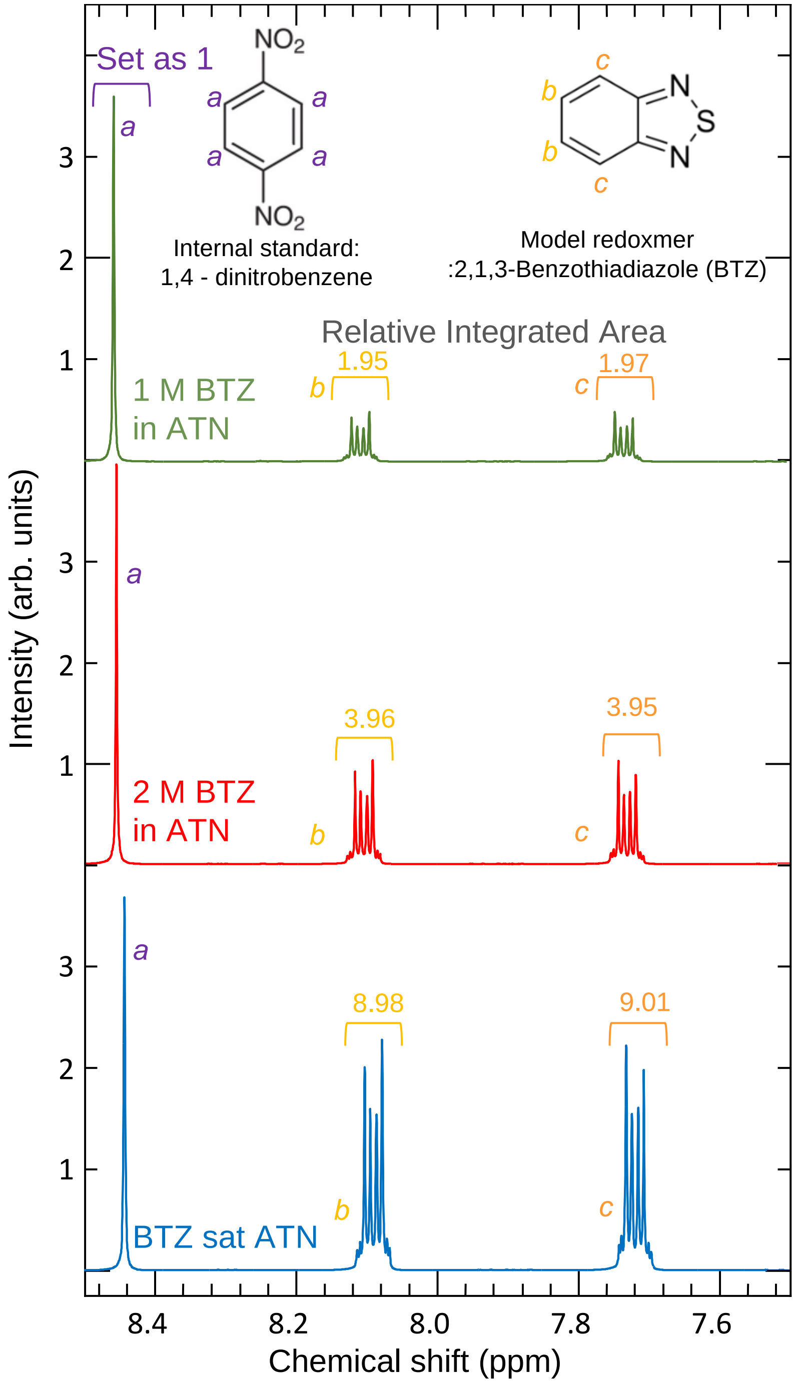

Fig. S7. ^{1}H-NMR spectra of 2,1,3-benzothiadiazole (BTZ) and 1,4-dinitrobenzene (DNB) for concentration quantification and their molecular structures.

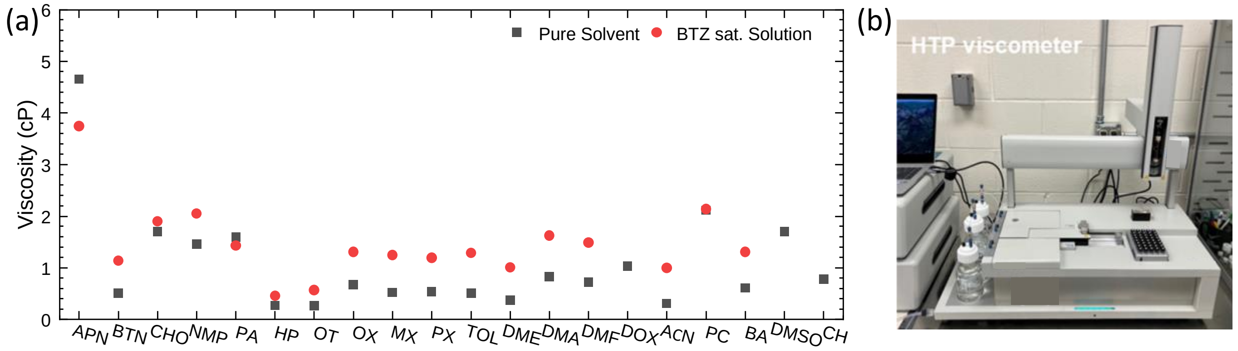

Fig. S8. (a) Steady shear viscosity as a function of solvent at 20 °C. (b) The high-throughput viscometer (VROC® initium one plus, RheoSense) used in this study. Source data are provided as a Source Data file.

Table. S1. Summary of processing time for Manual Excess Solute and HTE Excess Solute (this work).

		Manual Excess Solute	HTE Excess Solute	Automated Excess Solvent
Experimental runtime (min)	Programing	0	30	
	Solution Preparation	10	330	
	Stabilization	480	480	
	NMR Sampling	15	120	
	NMR Measurement	15	600	
	Post-Analysis	5	60	
	Total Process Time	525	1620	
Number of samples in a single batch		1	42	
Experimental runtime per sample (min/sample)		525	38.6	20-80*

*Shiri P, et al. iScience 24, 102176 (2021).

Table S2. Measured solubilities of BTZ in 22 single solvents at 20 ℃.

Solvent	Molarity (M, mol/L)
DME	4.93
DOX	5.47
ACN	4.27
DMF	5.39
DMA	5.21
PC	2.79
BA	4.17
DMSO	4.26
CH	2.06
TOL	4.79
HP	0.96
OT	0.97
PA	1.57
PX	4.40
MX	4.49
OX	4.57
NMP	5.48
CHO	5.09
HMPA	4.49
BTN	4.20
APN	2.28
GTN	1.86

Table S3. Measured solubilities of BTZ in 36 binary solvents at 20 °C.

Solvent 1	Volume fraction 1	Solvent 2	Volume fraction 2	Molarity (M)
DME	0.5	CH	0.5	4.3
PC	0.5	DMSO	0.5	3.59
BA	0.5	CH	0.5	3.7
PC	0.5	BA	0.5	4.05
DME	0.5	PC	0.5	4.13
ACN	0.5	DMA	0.5	4.87
DMA	0.5	PC	0.5	4.2
ACN	0.5	DMSO	0.5	4.26
PC	0.5	CH	0.5	4.28
BA	0.5	DMSO	0.5	4.76
ACN	0.5	BA	0.5	4.56
DOX	0.5	CH	0.5	4.88
DME	0.5	BA	0.5	4.59
DOX	0.5	PC	0.5	4.63
DME	0.5	DMSO	0.5	5.03
DMSO	0.5	CH	0.5	4.68
ACN	0.5	DMF	0.5	4.86
DMF	0.5	DMSO	0.5	4.83
DMA	0.5	CH	0.5	4.84
ACN	0.5	PC	0.5	3.43
DMF	0.5	PC	0.5	4.14
DOX	0.5	BA	0.5	4.88
DME	0.5	DMF	0.5	5.4
DMA	0.5	DMSO	0.5	4.9
ACN	0.5	CH	0.5	4.64
DOX	0.5	DMA	0.5	5.59
DMA	0.5	BA	0.5	5.1
DMF	0.5	CH	0.5	5.24
ACN	0.5	DOX	0.5	5.3
DME	0.5	DMA	0.5	5.3
DOX	0.5	DMSO	0.5	5.37
DMF	0.5	DMA	0.5	5.37
ACN	0.5	DME	0.5	4.58
DME	0.5	DOX	0.5	5.44
DMF	0.5	BA	0.5	5.07
DOX	0.5	DMF	0.5	5.67

Table S4. List of 11 features used in GPR models and Bayesian optimization workflow

No.	Feature name	Type	Feature creation
1	Topological surface area	Solvent, Molecular	RDKit
2	Molecular weight	Solvent, Molecular	RDKit
3	Number of heavy atoms	Solvent, Molecular	RDKit
4	Octanol/water partition coefficient (logP)	Solvent, Molecular	RDKit
5	Solvation free energies	Solute in continuum model, Electronic	Gaussian 16
6	Dipole moment	Solute, Electronic	Gaussian 16
7	Polarizability	Solute, Electronic	Gaussian 16
8	HOMO energy	Solute, Electronic	Gaussian 16
9	LUMO energy	Solute, Electronic	Gaussian 16
10	Maximum partial charge	Solute, Electronic	Gaussian 16
11	Minimum partial charge	Solute, Electronic	Gaussian 16

Table S5. Measured solubilities of BTZ in 40 binary solvents at 20 °C (Tested set in Fig. 3a).

Sample No.	Solvent 1	Volume fraction 1	Solvent 2	Volume fraction 2	Molarity (M)
046-1	OT	0.4	HMPA	0.6	4.31
046-2	ACN	0.1	CH	0.9	3.64
046-3	BA	0.7	HP	0.3	3.81
046-4	NMP	0.8	GTN	0.2	5.37
046-5	HP	0.3	CHO	0.7	4.96
046-6	PX	0.2	HMPA	0.8	5.24
046-7	CH	0.9	HP	0.1	2.07
046-8	ACN	0.3	CH	0.7	5.09
046-9	OT	0.6	HMPA	0.4	3.82
046-10	DOX	0.6	DMF	0.4	6.25
046-11	BA	0.8	PX	0.2	4.61
046-12	OT	0.7	HMPA	0.3	0.00
046-13	PX	0.1	HMPA	0.9	5.23
046-14	BA	0.1	OT	0.9	1.52
046-15	HP	0.2	OX	0.8	4.47
046-16	HMPA	0.4	APN	0.6	3.47
046-17	CHO	0.6	HMPA	0.4	5.54
046-18	DMA	0.2	HMPA	0.8	5.31
046-19	DME	0.8	OT	0.2	5.51
046-20	DME	0.7	OX	0.3	5.99
046-21	BA	0.7	APN	0.3	4.38
046-22	DMSO	0.1	HMPA	0.9	5.16
046-23	CH	0.9	PA	0.1	3.31
046-24	HMPA	0.7	APN	0.3	4.35
046-25	OT	0.3	HMPA	0.7	4.62
046-26	OT	0.5	HMPA	0.5	3.98
046-27	ACN	0.4	GTN	0.6	2.89
046-28	DME	0.3	HMPA	0.7	5.23
046-29	DMF	0.2	DMA	0.8	5.95
046-30	HMPA	0.4	BTN	0.6	4.94
046-31	ACN	0.4	HP	0.6	4.71
046-32	OT	0.5	PA	0.5	2.23
046-33	DOX	0.2	OX	0.8	5.48
046-34	ACN	0.7	CH	0.3	5.61
046-35	DOX	0.8	APN	0.2	6.21
046-36	DME	0.1	HMPA	0.9	5.47
046-37	PX	0.6	APN	0.4	4.57
046-38	OT	0.8	HMPA	0.2	2.78

046-39	ACN	0.3	OT	0.7	4.13
046-40	TOL	0.5	HMPA	0.5	5.24

Table S6. List of BO-suggested solvents and their measured solubilities in the 1st batch at 20 ℃.

Sample No.	Solvent 1	Volume fraction 1	Solvent 2	Volume fraction 2	Molarity (M)
326-01	DME	0.1	DOX	0.9	6.07
326-02	DOX	0.9	HMPA	0.1	6.12
326-03	DME	0.2	DOX	0.8	6.01
326-04	DME	0.3	DOX	0.7	5.98
326-05	DOX	0.9	BA	0.1	5.90
326-06	DOX	0.8	HMPA	0.2	5.97
326-07	DOX	0.9	DMF	0.1	5.94
326-08	DOX	0.9	NMP	0.1	6.09
326-09	DOX	0.9	DMA	0.1	5.49
326-10	DME	0.4	DOX	0.6	5.87
326-11	DOX	0.9	OX	0.1	6.30
326-12	DOX	0.7	HMPA	0.3	5.66
326-13	DOX	0.9	MX	0.1	6.08
326-14	DOX	0.9	PX	0.1	6.19
326-15	DOX	0.9	DMSO	0.1	5.58
326-16	DOX	0.9	TOL	0.1	5.90
326-17	DME	0.5	DOX	0.5	5.70
326-18	DOX	0.8	BA	0.2	5.89
326-19	DOX	0.9	CHO	0.1	6.12
326-20	DOX	0.9	OT	0.1	5.74
326-21	DOX	0.9	PA	0.1	6.21
326-22	DME	0.6	DOX	0.4	5.81
326-23	DOX	0.8	DMF	0.2	6.10
326-24	DOX	0.9	PC	0.1	5.45
326-25	DOX	0.8	NMP	0.2	6.34
326-26	DOX	0.9	HP	0.1	6.03
326-27	DOX	0.6	HMPA	0.4	5.56
326-28	DOX	0.9	CH	0.1	6.01
326-29	DOX	0.8	DMA	0.2	5.99
326-30	DOX	0.7	BA	0.3	5.96
326-31	DME	0.7	DOX	0.3	5.82
326-32	DOX	0.9	APN	0.1	5.42
326-33	DOX	0.8	OX	0.2	6.14
326-34	DOX	0.9	GTN	0.1	6.48

326-35	DOX	0.8	DMSO	0.2	6.50
326-36	DOX	0.9	BTN	0.1	4.53
326-37	DOX	0.8	MX	0.2	6.07
326-38	DOX	0.8	PX	0.2	6.03
326-39	DOX	0.5	HMPA	0.5	5.69
326-40	DOX	0.8	TOL	0.2	6.45

Table S7. List of BO-suggested solvents and their measured solubilities in the 2nd batch at 20 °C.

Sample No.	Solvent 1	Volume fraction 1	Solvent 2	Volume fraction 2	Molarity (M)
337-01	DOX	0.4	BA	0.6	5.66
337-02	DOX	0.5	BA	0.5	5.78
337-03	DOX	0.3	NMP	0.7	6.38
337-04	DOX	0.4	NMP	0.6	6.38
337-05	DOX	0.3	BA	0.7	5.55
337-06	DOX	0.2	NMP	0.8	5.98
337-07	DOX	0.5	NMP	0.5	6.37
337-08	DOX	0.6	CHO	0.4	6.11
337-09	DOX	0.4	OX	0.6	5.94
337-10	DOX	0.5	OX	0.5	5.97
337-11	DOX	0.5	CHO	0.5	6.34
337-12	DOX	0.3	DMA	0.7	6.00
337-13	DOX	0.4	MX	0.6	5.87
337-14	DOX	0.4	PX	0.6	5.90
337-15	DMF	0.6	HMPA	0.4	5.65
337-16	DOX	0.4	DMA	0.6	6.16
337-17	DME	0.8	DOX	0.2	5.87
337-18	DOX	0.6	APN	0.4	5.43
337-19	DOX	0.5	PC	0.5	5.38
337-20	DOX	0.5	MX	0.5	5.94
337-21	DOX	0.5	PX	0.5	5.89
337-22	DMF	0.5	HMPA	0.5	5.06
337-23	DOX	0.5	APN	0.5	4.96
337-24	DOX	0.6	GTN	0.4	5.34
337-25	DMF	0.7	HMPA	0.3	5.67
337-26	DOX	0.6	BA	0.4	5.92
337-27	DME	0.7	HMPA	0.3	5.70
337-28	DOX	0.5	GTN	0.5	4.86
337-29	DOX	0.2	DMA	0.8	6.13
337-30	DOX	0.4	PC	0.6	4.93

337-31	DME	0.6	HMPA	0.4	5.66
337-32	DOX	0.3	HMPA	0.7	5.79
337-33	DOX	0.6	PC	0.4	5.65
337-34	DOX	0.5	DMA	0.5	6.45
337-35	DME	0.8	HMPA	0.2	5.84
337-36	DOX	0.2	BA	0.8	5.34
337-37	DME	0.9	DOX	0.1	6.40
337-38	DME	0.4	NMP	0.6	6.35
337-39	DME	0.4	DMF	0.6	6.16
337-40	DOX	0.6	NMP	0.4	6.27

Table S8. List of BO-suggested solvents and their measured solubilities in the 3rd batch at 20 °C.

Sample No.	Solvent 1	Volume fraction 1	Solvent 2	Volume fraction 2	Molarity (M)
603-01	DME	0.7	OX	0.3	5.72
603-02	DOX	0.6	DMSO	0.4	5.97
603-03	DOX	0.7	ACN	0.3	5.92
603-04	DME	0.7	PX	0.3	5.36
603-05	DME	0.7	MX	0.3	5.44
603-06	DME	0.8	OX	0.2	5.60
603-07	DME	0.8	MX	0.2	5.43
603-08	DME	0.6	OX	0.4	5.54
603-09	DME	0.8	PX	0.2	5.64
603-10	DME	0.8	TOL	0.2	5.53
603-11	DOX	0.5	DMSO	0.5	6.10
603-12	DME	0.9	TOL	0.1	5.66
603-13	DOX	0.5	DMF	0.5	6.26
603-14	DME	0.6	MX	0.4	5.32
603-15	DME	0.6	PX	0.4	5.28
603-16	DOX	0.4	DMF	0.6	5.98
603-17	DME	0.9	MX	0.1	5.46
603-18	DME	0.9	PX	0.1	5.05
603-19	DME	0.9	OX	0.1	5.08
603-20	DOX	0.8	ACN	0.2	6.20
603-21	DOX	0.7	DMSO	0.3	6.02
603-22	DOX	0.6	ACN	0.4	6.04
603-23	DME	0.7	TOL	0.3	5.31
603-24	DOX	0.6	PA	0.4	5.61
603-25	DME	0.9	DMSO	0.1	5.67

603-26	DME	0.9	CH	0.1	5.65
603-27	DME	0.9	HP	0.1	5.53
603-28	DME	0.9	OT	0.1	5.21
603-29	DME	0.8	DMSO	0.2	5.74
603-30	DME	0.8	DMF	0.2	5.85
603-31	DME	0.9	DMF	0.1	5.77
603-32	DME	0.7	DMF	0.3	5.80
603-33	DOX	0.5	PA	0.5	5.46
603-34	DOX	0.3	DMF	0.7	6.14
603-35	DME	0.9	DMA	0.1	5.71
603-36	DOX	0.4	DMSO	0.6	5.88
603-37	DOX	0.7	PA	0.3	6.14
603-38	DME	0.6	DMF	0.4	5.95
603-39	DME	0.8	DMA	0.2	5.74
603-40	DME	0.9	NMP	0.1	5.86

Table S9. Stabilization time comparison after mixing the solutions.

Solvent	Molarity (M, mol/L at 20 °C)		
	6 hours	8 hours	20 hours
DOX	5.50	5.47	5.48
PC	2.77	2.79	2.78
DMSO	4.30	4.26	4.31
CH	2.08	2.06	2.09
ACN	4.77	4.64	4.8
