# Supplementary material for: An integrated high-throughput robotic platform and active learning approach for accelerated discovery of optimal electrolyte formulations
Source: Nat Commun. 2024 Mar 29;15:2757. doi: 10.1038/s41467-024-47070-5 (PMC10980761; doi:10.1038/s41467-024-47070-5)
Supplement: Supplementary file 3 — Description of Additional Supplementary Files [file 41467_2024_47070_MOESM3_ESM.pdf]

Description of Additional Supplementary Files

File Name: Supplementary Movie 1

Description: With the help of an automated system, BTZ powder was accurately dispensed into individual vials housed in a 48-vial plate. This facilitated precise, safe, and efficient experimental processes.

File Name: Supplementary Movie 2

Description: Using an automated high-resolution camera system, images of the saturated solutions were captured and stored automatically.
